# Educational intervention with a booklet on syphilis for women in situations of sexual vulnerability: a quasi-experimental study^1^

**DOI:** 10.1590/1518-8345.8010.4807

**Published:** 2026-03-16

**Authors:** Vanessa Kelly Cardoso Estumano, Pedro Paulo Santos Nunes, Aline Maria Pereira Cruz Ramos, Marcia Helena Machado Nascimento, Guilherme Guarino de Moura Sá, Eliã Pinheiro Botelho, Cintia Yolette Urbano Pauxis Aben-Athar

**Affiliations:** 1 Universidade Federal do Pará, Belém, PA, Brazil.; 2 Universidade Federal do Pará, Faculdade de Enfermagem, Belém, PA, Brazil.; 3 Scholarship holder at the Coordenação de Aperfeiçoamento de Pessoal de Nível Superior (CAPES), Brazil.; 4 Universidade do Estado do Pará, Belém, PA, Brazil.; 5 Instituto Federal de Educação, Ciência e Tecnologia de Pernambuco, Pesqueira, PE, Brazil.

**Keywords:** Women, Vulnerable Populations, Sexual Vulnerability, Syphilis, Intervention Study, Nursing.

## Abstract

**Objective:**

to assess riverside women’s knowledge about syphilis before and after a booklet educational intervention.

**Method:**

a quasi-experimental, comparative study with a before-and-after design was conducted with 40 riverside women through a discussion group with shared reading of the booklet. The nonparametric Wilcoxon test was used for data analysis.

**Results:**

most women were between 18 and 29 years old, married or in a stable relationship, and had completed elementary school. Most had heard about syphilis from health professionals. They had been tested for syphilis and had previously undergone syphilis treatment. Comparing the pre-test and immediate post-test scores, an increase in knowledge about syphilis was observed. When analyzing the immediate post-test phase with the 7-day post-test, there was no significant difference; that is, the knowledge acquired remained the same after 7 days of the educational intervention. When analyzing the pre-test phase and the 7-day post-test phase, an improvement in knowledge was observed.

**Conclusion:**

the educational intervention through a booklet discussion circle with shared reading proved effective in acquiring knowledge about syphilis among riverside women, evidenced by the significant improvement in knowledge in the post-test results.

## Highlights

**(1)** The women showed increased and maintained knowledge about syphilis. **(2)** The intervention with the booklet and discussion group was effective in increasing knowledge. **(3)** The booklet is a technology that facilitates the teaching-learning process. **(4)** The discussion group with shared reading of the booklet increased knowledge.

## Introduction

Syphilis is a sexually transmitted infection (STI) considered a public health problem because it is a global condition^1^. Despite advances in access to diagnosis through rapid testing, which facilitates identification and treatment, a reduction in cases of the disease has not yet been observed, which points to gaps in control and prevention strategies^2^.

Given this scenario, the persistence of syphilis cases highlights the urgent need for prevention actions to achieve the goals established in the United Nations (UN) 2030 Agenda for STI reduction, including syphilis^3^ ^-^ ^4^. In this context, initiatives to combat syphilis must go beyond diagnosis and treatment and must be grounded in social movements and health planning^5^. It is essential that these actions be specifically targeted at vulnerable communities and populations that face limited access to health care.

This difficulty in accessing characteristic of populations such as riverside communities, is amplified in vast and complex Brazilian territories, such as the Amazon. In these regions, vast geographic distances and social inequality perpetuate the transmission of syphilis among adults^2^ ^,^ ^6^, with a lack of knowledge about STIs identified as one of the factors contributing to infection by *Treponema pallidum*, the etiological agent of the disease^2^.

Within this context of heightened vulnerability, the riverside communities of the Amazon, the focus of this study, have their daily lives shaped by the influence of rivers. Their economy, based on fishing, plant extraction, and sometimes tourism, coexists with precarious infrastructure. This reality is marked by a lack of basic sanitation, limited access to electricity, and restricted access to health services and public programs^6^ ^-^ ^7^.

Although characterized by the centrality of women and their essential role in shaping the community, riverside women in the Amazon face significant social vulnerabilities^8^. This scenario, combined with low knowledge and risky sexual behaviors, makes them particularly susceptible to adverse health outcomes, especially regarding sexual health^6^ ^,^ ^9^ ^-^ ^10^.

Therefore, the lifestyle and context of these populations require a differentiated response to syphilis, distinct from the approach for the general population. Therefore, these vulnerable populations must have access to quality information through health promotion and syphilis prevention actions carried out by the healthcare team^6^.

For these actions to be truly effective, it is imperative that they address regional peculiarities, as well as the behaviors and attitudes^2^ of residents of different vulnerable contexts in Brazil, whether rural, riverside, or forested. The healthcare team must, therefore, conduct health education activities, in groups or individually, using accessible language and targeting local behavioral characteristics^10^.

In this sense, the use of validated and easily accessible technologies, such as booklets, is essential. They not only empower individuals but also allow them to act as multipliers, presenting the material to others in the community. Thus, booklets are useful materials for describing health-related topics and can be used as a health promotion tool, facilitating the educational process, and improving knowledge^11^.

Given the need for health education strategies for vulnerable populations and recognizing the effectiveness of validated educational technologies, this study aimed to assess the knowledge of riverside women about syphilis before and after an educational intervention using a booklet.

## Method

### Study design

This is a quasi-experimental, comparative study with a pre- and post-test design. Participants were assessed before and after the educational activity. The study follows the Transparent Reporting of Evaluations with Nonrandomized Designs (TREND) checklist^12^.

This study is part of the macro-project “Situational Diagnosis of Sexually Transmitted Infections in the Amazon Context”. The research was based on two previous stages of the project: 1. Development and Validation of a Booklet in a previous study^13^: The educational booklet “Let’s Talk About Syphilis” was developed using language and expressions from the riverside community to address transmission, symptoms, diagnosis, treatment, and prevention^14^. 2. Development and Validation of the Questionnaire^15^ “Assessment of Riverside Women’s Knowledge about Syphilis”, which is in the process of being published. Both materials underwent semantic validation with riverside women from Cotijuba Island, Belém, Pará, and content validation by experts.

Thus, the present study implemented the educational intervention through a booklet discussion circle with shared reading. 

### Place and period of study

Conducted in the Family Health Strategy (FHS) unit on Cotijuba Island, located near the city of Belém, in the state of Pará, in Brazil. It is approximately 22 km from the capital, Belém, and its main access route is via the river. The island has an estimated population of 9,000 inhabitants and occupies an area of 16 km^2^. The island’s population lives off commercial activities, artisanal fishing, family farming, agroextractivism, and tourism^16^. The study took place between April and May 2024.

### Participants

The study population consisted of riverside women from Cotijuba Island, Belém, Pará.

### Selection criteria

Riverside women aged 18 years or older, with no upper age limit, who were permanent residents of Cotijuba Island, and literate. The inclusion criterion for literate participants was self-declaration, followed by proof of the ability to read and sign the Informed Consent Form (ICF) at the time of recruitment. The exclusion criteria were women visiting or working non-residents of Cotijuba Island. 

### Sample definition

The women were selected according to demand, representing a consecutive sample during a health intervention carried out by nurses from the FHS on Cotijuba Island. The sample size was estimated at 40 women using the McNemar chi-square test, used in a previous study^11^. No woman declined to participate in the study.

The following parameters were adopted for the calculation: a 95% confidence interval, 80% statistical power, a 50% proportion of pairs that would not change with the implementation of the educational intervention (this value was adopted because this parameter was unknown), and a change in proportion of at least 20% between pairs of observations to reject the null hypothesis (i.e., no difference between the proportions before and after the implementation of the educational intervention), where Zα = 1.96; Zβ = 80%; PA = 0.2; qA = 0.8; PD = 0.5. After calculations, the sample was estimated at 40 participants.

### Instrument used to collect information

The validated questionnaire called “Assessment of Riverside Women’s Knowledge about Syphilis”^15^ developed by the researcher as part of her dissertation was used. The questionnaire consists of open-ended and closed-ended questions and is structured in three parts: sociodemographic data (age, education, and marital status), six dichotomous questions about behavioral factors, and ten questions about knowledge about syphilis. This last section is subdivided into four main topics: general aspects of the infection, diagnosis and prevention, treatment, and gestational and congenital syphilis.

### Data collection

The study was conducted in four sequential stages: (i) Pre-test, (ii) Educational intervention with a booklet “Let’s Talk About Syphilis” discussion circle and shared reading, (iii) Immediate post-test, and (iv) 7-day post-test. The activities were conducted in secure locations previously agreed upon with the participants, ensuring a safe and confidential environment for discussion and experience sharing.

Initially, 40 women were invited to participate in the study at the FHS. At this point, the researcher had asked about the women’s level of education to assess their ability to read the educational material. After signing the informed consent form, the participants completed a printed questionnaire (stage i), lasting 20 minutes, to assess their prior knowledge about syphilis and characterize their sociodemographic profile. Participants were instructed to answer the questionnaire based on their prior knowledge of syphilis and that they could not ask any questions or share their responses with others. Each questionnaire was coded to ensure traceability of responses.

In stage 2, each participant received a printed copy of the booklet “Let’s Talk About Syphilis” and was instructed to leaf through it and read it individually for 15 minutes. The nurse researcher then led a discussion group, where participants shared reading and handling of the booklet, as well as clarified any questions. The total duration of the discussion group was 30 minutes per group, with the same standardized duration for all three groups. It is worth noting that, during the discussion group, some women felt comfortable and shared their own experiences and those of friends/family members who had been diagnosed and treated for syphilis.

In stage 3, immediately after the intervention, the same questionnaire was administered again to assess the knowledge acquired (immediate post-test), with 20 minutes to complete. At the end of this stage, the booklets were given to the participants for home consultation. Finally, stage IV consisted of reapplication of the questionnaire via telephone contact, seven days after the intervention^11^ ^,^ ^17^, with the aim of verifying knowledge retention. Only one participant was not located, resulting in a sample of 39 women for this phase of the study.

### Data processing and analysis

The collected data were stored in a Microsoft Excel^®^ 2019 spreadsheet, and statistical analysis was performed using BioEstat^®^ version 5.0 and Minitab^®^ software. Initially, a descriptive analysis of the sociodemographic characteristics and behavioral factors of the riverside women was performed, described using frequencies and percentages.

Knowledge data were initially analyzed using descriptive statistics, with absolute and relative frequencies and measures of central tendency. To assess whether there was a difference in scores between the groups before and after the intervention, the Shapiro-Wilk test was used to identify the normality of the variables. After the results demonstrated a non-normal distribution, the non-parametric Wilcoxon test was used to compare the riverside women’s knowledge in the pre-test, immediate post-test, and 7-day post-test phases. A p-value ≤ 0.05 was considered significant. A box plot was used to demonstrate the analysis of statistical significance between the groups*.*

### Ethical considerations

This study is part of the final stage of a macroproject approved by the Research Ethics Committee of the Institute of Health Sciences (ICS) of the Federal University of Pará (UFPA) under CAAE No. 10821819.0.0000.0018 and opinion No. 6,301,468. Written informed consent was obtained from all participants involved in the study. 

## Results

Regarding sociodemographic data, there was a prevalence of riverside women aged 18 to 29 (n=14; 35.0%), married and/or in a stable union (n=23; 57.5%), with complete elementary education (n=21; 52.5%).

Most women had heard about syphilis (n=37; 92.5%) from healthcare professionals (n=25; 67.6%) or attended lectures (n=23; 57.5%). They had already been tested (n=27; 67.5%) for syphilis diagnosis at some point, with the rapid test (n=14, 51.9%) being the most frequently cited method. Previous treatment for syphilis was also reported (n=14, 35.0%) and non-use of condoms during sexual intercourse (n=23, 57.5%). Regarding the assessment of syphilis knowledge, Table 1 presents the number of correct answers regarding the riverside women’s knowledge about syphilis in the pre-test, immediate post-test, and 7-day post-test, considering a total of 10 questions per test. It is noteworthy that only 39 women participated in the 7-day post-test phase. These data show that there was a greater number of correct answers on the topic in both post-test moments when compared to the pre-test conducted before the educational intervention using the booklet. 

When comparing the measures of central tendency of the women’s correct answers, it was observed that the mean in the pre-test was 6.25 (Median: 6.0; Standard Deviation: 1.46; Minimum: 3.0 and Maximum: 9.0), in the immediate post-test it was 8.95 (Median: 9.0; Standard Deviation: 0.87; Minimum: 7.0 and Maximum: 10.0) and in the 7-day post-test it was 8.89 (Median: 9.0; Standard Deviation: 0.94; Minimum: 7.0 and Maximum: 10.0). Therefore, it is worth highlighting the improvement in the acquisition and retention of knowledge about syphilis after the educational intervention. Knowledge about syphilis according to the dimensions of analysis in the immediate post-test, the median was higher than in the pre-test, with statistical significance in all dimensions (p ≤ 0.05), according to Table 2.

When comparing knowledge about syphilis, according to the analysis dimensions, between the two post-test moments, it was observed that there was knowledge retention even after 7 days of intervention, as shown in Table 3. 

Table 4 shows the comparison of knowledge about syphilis in the pre-test and 7-day post-test according to the dimensions of analysis. Statistical significance is observed in all dimensions, which suggests an increase in knowledge of such aspects.

**Figure 1:**
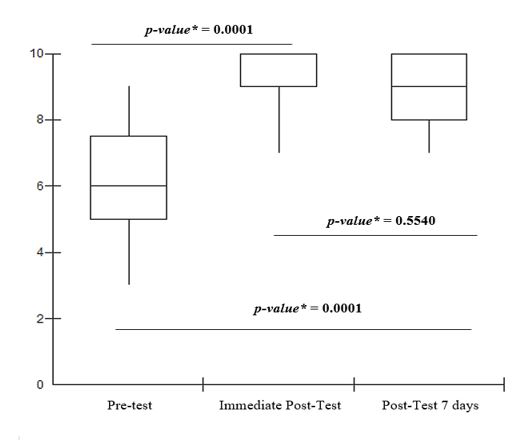
Analysis of statistical significance between groups in the pre-test, immediate post-test and 7-day post-test

Riverside women who participated in the educational intervention on syphilis with the booklet showed an increase in their knowledge about syphilis (p = 0.0001) in the immediate post-test (Figure 1). When analyzing the immediate post-test with the 7-day post-test, it was observed that the acquired knowledge about syphilis was maintained for 7 days. When comparing the pre-test and the 7-day post-test, improvement and retention of knowledge were observed (p = 0.0001).

**Table 1:** Number of correct answers of riverside women regarding knowledge about syphilis, in the pre-test (n = 40), immediate post-test (n = 40), and 7-day post-test (n = 39) moments. Belém, PA, Brazil, 2024

Riverside woman	Pre-Test		Immediate Post-Test		Post-Test 7 days	
	n	%	n	%	n	%
1	5	50	10	100	10	100
2	6	60	10	100	10	100
3	5	50	9	90	8	80
4	3	30	9	90	10	100
5	7	70	7	70	9	90
6	8	80	8	80	8	80
7	8	80	10	100	10	100
8	7	70	8	80	8	80
9	5	50	9	90	8	80
10	6	60	9	90	9	90
11	3	30	8	80	8	80
12	8	80	9	90	10	100
13	8	80	9	90	8	80
14	8	80	9	90	8	80
15	9	90	10	100	10	100
16	6	60	7	70	7	70
17	6	60	10	100	10	100
18	5	50	8	80	8	80
19	7	70	8	80	9	90
20	5	50	8	80	8	80
21	7	70	10	100	9	90
22	6	60	9	90	9	90
23	7	70	9	90	9	90
24	7	70	9	100	10	100
25	6	60	9	90	9	90
26	5	50	9	100	10	10
27	7	70	9	100	9	90
28	8	80	8	80	8	80
29	5	50	10	100	10	100
30	8	80	9	100	8	80
31	5	50	9	90	9	90
32	5	50	9	90	8	80
33	6	70	10	100	10	100
34	6	60	10	100	7	70
35	7	70	9	90	9	90
36	4	40	9	90	8	80
37	5	50	10	100	9	90
38	8	80	9	90	10	100
39	8	80	10	100	10	100
40	8	80	10	100	-	-

**Table 2:** Comparison of knowledge about syphilis among riverside women (n = 40) in the pre-test and immediate post-test according to each question of the questionnaire. Belém, PA, Brazil, 2024

Quiz questions	Pre-Test		Immediate Post-Test		p-value*
	n	%	n	%	
**General aspects of syphilis infection**					
1. Etiology	34	87.1	40	100	
2. Syphilis Transmission	35	89.7	40	100	
3. Signs and Symptoms	27	69.2	34	85.0	
Median total score for general aspects	2		3		<0.0001
**Diagnosis and prevention**					
4. Diagnosis	23	57.5	34	85.0	
5. Prevention	30	76.9	37	92.5	
Median total diagnosis and prevention score	1		2		<0.0001
**Treatment**					
6. Appropriate Treatment	29	72.5	37	92.5	
7. Partner Treatment	23	59.0	33	82.5	
Median total score for treatment questions	1		2		0.002
**Gestational and congenital syphilis**					
8. Diagnosis of Gestational Syphilis	20	51.3	34	85.0	
9. Complications of Gestational Syphilis	25	62.5	37	92.5	
10. Complications of Congenital Syphilis	27	69.2	36	90.0	
Median total score for gestational and congenital syphilis	2		3		<0.0001

*p-value - Wilcoxon test

**Table 3:** Comparison of riverside women’s knowledge about syphilis in the immediate post-test (n = 40) and 7-day post-test (n = 39) according to each question of the questionnaire. Belém, PA, Brazil, 2024

Quiz questions	Immediate Post-Test		Post-Test 7 days		p-value*
	n	%	n	%	
**General aspects of syphilis infection**				
1. Etiology	40	100	39	100	
2. Syphilis Transmission	40	100	39	100	
3. Signs and Symptoms	34	85.0	32	82.1	
Median total score for general aspects	3		3		0.425
**Diagnosis and prevention**					
4. Diagnosis	34	85.0	36	92.3	
5. Prevention	37	92.5	36	92.3	
Median total score for diagnosis and prevention	2		2		0.563
**Treatment**					
6. Appropriate Treatment	37	92.5	31	79.5	
7. Partner Treatment	33	82.5	34	87.2	
Median total treatment score	2		2		0.463
**Gestational and congenital syphilis**					
8. Diagnosis of Gestational Syphilis	34	85.0	35	89.7	
9. Complications of Gestational Syphilis	37	92.5	36	92.3	
10. Complications of Congenital Syphilis	36	90.0	38	97.4	
Median total score for gestational and congenital syphilis		3	3		0.207

*p-value - Wilcoxon test

**Table 4:** Comparison of riverside women’s knowledge about syphilis in the pre-test (n = 40) and 7-day post-test (n = 39) according to each question of the questionnaire. Belém, PA, Brazil, 2024

Quiz questions	Pre-Test		Post-Test 7 days		p-value*
	n	%	n	%	
**General aspects of syphilis infection**				
1. Etiology	34	87.1	39	100	
2. Syphilis Transmission	35	89.7	37	94.9	
3. Signs and Symptoms	27	69.2	32	82.1	
Median total score for general aspects	2		3		<0.0001
**Diagnosis and prevention**				
4. Diagnosis	22	56.4	36	92.3	
5. Prevention	30	76.9	36	92.3	
Median total diagnosis and prevention score	1		2		<0.0001
**Treatment**				
6. Appropriate Treatment	28	71.8	31	7.5	
7. Partner Treatment	22	56.4	34	87.2	
Median total treatment score	1		2		0.015
**Gestational and congenital syphilis**				
8. Diagnosis of Gestational Syphilis	20	51.3	35	89.7	
9. Complications of Gestational Syphilis	24	61.5	36	92.3	
10. Complications of Congenital Syphilis	27	69.2	38	97.4	
Median total score for gestational and congenital syphilis	2		3		<0.0001

*p-value - Wilcoxon test

*p-value - Wilcoxon test

## Discussion

The women participating in the educational intervention, young, with elementary education, and married or in a stable relationship, reflect the profile of other riverside communities in Brazil^6^ ^,^ ^18^. The limited prior knowledge identified in the pre-test among participants (median = 6 correct answers), combined with non-use of condoms, highlights a behavioural vulnerability that increases the risk of acquiring STIs.

It is known that inadequate or absent use of condoms is a factor of individual vulnerability that should be addressed by health teams through educational technologies and accessible language^10^ ^,^ ^19^. Furthermore, although some knew how to answer some questions about the disease, they were unable to respond to more specific questions, reinforcing the need for greater investment in health education.

After the educational intervention, the median number of correct answers increased significantly to 9 in the immediate post-test and remained unchanged in the 7-day post-test. This knowledge acquisition is essential for the prevention and adherence to syphilis treatment, as a lack of knowledge can intensify the disease’s problems, hindering prevention and cure^11^.

The effectiveness of the educational intervention with discussion circles and shared reading of the booklet was confirmed by comparing the pre-test and immediate post-test, with an increase in the number of correct answers to the questions, highlighting the improvement in knowledge about syphilis. A quasi-experimental study^11^, which evaluated the effects of knowledge, attitudes, and practices of pregnant women before and after an educational intervention involving the reading of a booklet on gestational syphilis, identified a statistically significant improvement in these three domains after reading the material. Other quasi-experimental studies^20^ ^-^ ^22^ found an increase in the number of correct answers to questions and the level of knowledge about a given disease after an educational intervention with group activities and an educational video.

Our findings are in line with international studies that have also verified the effectiveness of educational interventions in vulnerable contexts. In Mozambique, a randomized controlled clinical trial with women at risk of HIV/AIDS used a psychosocial educational intervention that resulted in a significant increase in knowledge about the risk factors of the disease and greater adherence to preventive practices^23^.

In rural areas of Peru, interventions with family nutrition education programs demonstrated a positive effect on knowledge levels and the adoption of healthier eating habits^24^. These results, across different geographic contexts, reinforce that educational interventions are powerful tools for promoting health in vulnerable communities.

Although the follow-up period was short (7 days), the maintenance of knowledge about syphilis after the intervention suggests that the booklet has the potential to promote information retention, which is a promising result. A study that aimed to evaluate the effectiveness of an educational intervention on treatment adherence and knowledge about syphilis in postpartum women who tested positive for the disease found that the post-test revealed improvements in knowledge about syphilis, with a 30-day post-test score associated with better treatment adherence^25^. Another study on syphilis in pregnant women found an increase in knowledge after the booklet intervention, both in the immediate post-test and on the 7th day^11^.

Vertical transmission of syphilis continues to be a public health challenge^26^, with barriers such as unfavourable social conditions and lack of knowledge^27^. Therefore, educational initiatives aimed at vulnerable populations are effective strategies to encourage the adoption of health promotion practices and contribute to greater disease control^28^.

Therefore, the implementation of strategies such as the use of accessible technologies and educational interventions have proven effective in improving knowledge and health care^11^ ^,^ ^19^ ^,^ ^25^. Multimodal educational interventions, with validated methodology and materials, are believed to improve knowledge and self-care, especially among the most vulnerable^29^.

It is reiterated that the educational intervention has been shown to be effective in improving knowledge about syphilis among riverside women, with positive results maintained for 7 days. However, the persistence of some errors in the post-test suggests that learning is an ongoing process and that periodic interventions are necessary. Therefore, the role of the health team is crucial in establishing a bond with the community, facilitating the adoption of healthy behaviours and ensuring that the knowledge acquired translates into concrete prevention and self-care actions, contributing to disease control^11^ ^,^ ^25^ ^,^ ^27^. 

In practice, the booklet intervention proved most effective when conducted in a discussion circle format with shared reading. This approach facilitated interaction, allowing riverside women to feel comfortable sharing their experiences and clarifying questions, reinforcing the importance of a participatory method. The intervention was low-cost, and the effectiveness observed in this study justifies future research to explore its replication and adaptability in other contexts, both in rural and urban areas.

Limitations of the study include the small sample size of the educational intervention and its implementation in a single community, which limits its generalizability. Therefore, future studies that can expand the sample size of women from different riverside communities are important, as is a randomized controlled trial to identify the effect of the educational booklet on changing knowledge about syphilis.

The study’s main contribution is the scientific evidence for the effectiveness of nurse-mediated educational interventions, including discussion groups and shared reading of the booklet, in a specific population that has been understudied, particularly due to limited access. Furthermore, this not only improves health education strategies for vulnerable populations but also highlights nursing’s potential to lead and implement low-cost solutions with social impact, enabling adherence to preventive practices and addressing public health problems such as syphilis.

## Conclusion

The educational intervention, conducted in a discussion circle format with shared reading of the booklet “Let’s Talk About Syphilis”, proved effective in acquiring knowledge about syphilis among riverside women, as evidenced by the significant improvement in post-test results. The retention of knowledge for seven days suggests the booklet’s potential to act as a lasting support resource. The clear and accessible language of the material, combined with the dynamics of the discussion circle, proved to be a valuable tool for the teaching-learning process. The booklet has proven applicability and can be adapted and used by a variety of healthcare professionals in different contexts, both in rural communities with limited access and in urban areas, aiding in syphilis prevention and promoting self-care.

## Data Availability

Datasets related to this article will be available upon request to the corresponding author.
