# Supplementary material for: Educational intervention with a booklet on syphilis for women in situations of sexual vulnerability: a quasi-experimental study
Source: Rev Lat Am Enfermagem. 2026 Mar 16;34:e4807. doi: 10.1590/1518-8345.8010.4807 (PMC12991392; doi:10.1590/1518-8345.8010.4807)
Supplement: Translation

# Translation: Intervenção educativa com cartilha sobre sífilis para mulheres em situação de vulnerabilidade sexual: estudo quase-experimental^2^

**DOI:** 10.1590/1518-8345.8010.4808

**Authors:** Vanessa Kelly Cardoso Estumano, Pedro Paulo Santos Nunes, Aline Maria Pereira Cruz Ramos, Marcia Helena Machado Nascimento, Guilherme Guarino de Moura Sá, Eliã Pinheiro Botelho, Cintia Yolette Urbano Pauxis Aben-Athar

**Affiliations:** 1 Universidade Federal do Pará, Belém, PA, Brasil.; 2 Universidade Federal do Pará, Faculdade de Enfermagem, Belém, PA, Brasil.; 3 Bolsista da Coordenação de Aperfeiçoamento de Pessoal de Nível Superior (CAPES), Brasil.; 4 Universidade do Estado do Pará, Belém, PA, Brasil.; 5 Instituto Federal de Educação, Ciência e Tecnologia de Pernambuco, Pesqueira, PE, Brasil.

**Keywords:** Mulheres, Populações Vulneráveis, Vulnerabilidade Sexual, Sífilis, Estudo de Intervenção, Enfermagem.

## Abstract

**Objetivo:**

avaliar o conhecimento de mulheres ribeirinhas sobre sífilis antes e após intervenção educativa com uso de cartilha.

**Método:**

estudo quase-experimental, comparativo, com delineamento antes e depois, realizado com 40 mulheres ribeirinhas por meio de roda de conversa com leitura compartilhada da cartilha. Para análise dos dados, utilizou-se o teste não-paramétrico de Wilcoxon.

**Resultados:**

a maioria das mulheres tinha de 18 a 29 anos, era casada ou estava em união estável e tinha o ensino fundamental. A maioria já ouviu falar sobre sífilis por meio dos profissionais de saúde. Realizaram exame para detecção de sífilis e já realizaram tratamento anterior para sífilis. Ao comparar as pontuações do pré-teste e do pós-teste imediato, observou-se aumento do conhecimento sobre sífilis. Ao analisar a fase pós-teste imediato com o pós-teste 7 dias, não houve diferença significativa, ou seja, o conhecimento adquirido se manteve o mesmo após 7 dias da intervenção educativa. Ao analisar a fase pré-teste e a fase pós-teste 7 dias, observou-se melhora do conhecimento.

**Conclusão:**

a intervenção educativa por meio de roda de conversa com leitura compartilhada da cartilha mostrou-se eficaz na aquisição de conhecimento sobre sífilis entre mulheres ribeirinhas, evidenciada pela melhora significativa do conhecimento nos resultados do pós-teste.

## Destaques

**(1)** As mulheres apresentaram aumento e manutenção do conhecimento sobre sífilis. **(2)** A intervenção com a cartilha e roda de conversa foi eficaz no aumento do conhecimento. **(3)** A cartilha é uma tecnologia facilitadora do processo ensino-aprendizagem. **(4)** A roda de conversa com leitura compartilhada da cartilha aumentou o conhecimento.

## Introdução

A sífilis é uma Infecção Sexualmente Transmissível (IST) considerada como um problema de saúde pública por ser uma afecção de magnitude global^1^. Apesar dos avanços no acesso ao diagnóstico por meio de testes rápidos, que facilitam a identificação e o tratamento, a redução de casos da doença ainda não tem sido observada, o que aponta para lacunas nas estratégias de controle e prevenção^2^.

Diante desse cenário, a persistência dos casos de sífilis ressalta a necessidade urgente de ações de combate para que as metas estabelecidas na Agenda 2030 da Organização das Nações Unidas (ONU), quanto à redução de IST, entre elas a sífilis, sejam alcançadas^3^ ^-^ ^4^. Nesse contexto, as iniciativas de enfrentamento devem ir além do diagnóstico e tratamento, precisando ser fundamentadas no movimento social e no planejamento em saúde^5^. É fundamental que essas ações sejam especificamente direcionadas às comunidades e populações vulneráveis que enfrentam acesso dificultado à saúde. 

Essa dificuldade de acesso, característica de populações como as ribeirinhas, é amplificada nos vastos e complexos territórios brasileiros, como na Amazônia. Nessas regiões, as grandes distâncias geográficas e a desigualdade social perpetuam a transmissão da sífilis entre os adultos^2^ ^,^ ^6^, sendo a falta de conhecimento sobre as IST identificada como um dos fatores que contribuem para a infecção pelo *Treponema pallidum*, o agente etiológico da doença^2^. 

Dentro desse contexto de vulnerabilidade ampliada, as populações ribeirinhas da Amazônia, foco deste estudo, têm sua rotina moldada pela influência dos rios. A economia, baseada na pesca, no extrativismo vegetal e, por vezes, em atividades turísticas, convive com uma infraestrutura precária. Essa realidade é marcada pela falta de saneamento básico, acesso limitado à energia elétrica e acesso restrito a serviços de saúde e políticas públicas^6^ ^-^ ^7^.

Embora caracterizadas pela centralidade da presença feminina e pelo papel essencial da mulher na organização da comunidade, as mulheres ribeirinhas da Amazônia enfrentam vulnerabilidades sociais significativas^8^. Tal cenário, somado ao baixo conhecimento e comportamentos sexuais de risco, as torna particularmente suscetíveis a desfechos desfavoráveis à saúde, especialmente no que tange à saúde sexual^6^ ^,^ ^9^ ^-^ ^10^. 

Sendo assim, o estilo de vida e o cenário dessas populações exigem uma resposta diferenciada no enfrentamento da sífilis, distinta da abordagem para a população em geral. Logo, deve-se garantir o acesso dessas populações vulneráveis à informação de qualidade, por meio de ações de promoção à saúde e prevenção à sífilis realizadas pela equipe de saúde^6^. 

Para que essas ações sejam verdadeiramente eficazes, é imperativo que atendam às peculiaridades regionais, bem como aos comportamentos e atitudes^2^ dos moradores de diferentes contextos vulneráveis do Brasil, sejam eles dos campos, rios ou florestas. A equipe de saúde deve, portanto, realizar atividades de educação em saúde, em grupo ou individualmente, utilizando uma linguagem acessível e direcionada às características comportamentais locais^10^. 

Nesse sentido, o uso de tecnologias validadas e de fácil acesso, como as cartilhas, mostra-se fundamental, pois, além de auxiliar no empoderamento do indivíduo, permite que este atue como multiplicador, apresentando o material a outros indivíduos da comunidade. Dessa forma, as cartilhas são materiais úteis para descrição de assuntos relacionados à saúde, sendo possível sua utilização como instrumento de promoção da saúde, facilitador do processo educativo e melhoria do conhecimento^11^.

Tendo em vista a necessidade de estratégias de educação em saúde para populações vulneráveis, e reconhecendo a eficácia de tecnologias educativas validadas, este estudo teve como objetivo avaliar o conhecimento de mulheres ribeirinhas sobre sífilis antes e após intervenção educativa com uso de cartilha.

## Método

### Delineamento do estudo

Este é um estudo quase-experimental, comparativo, com delineamento antes e depois (pré e pós-teste). Os participantes serviram como seus próprios controles, sendo avaliados antes e depois da atividade educativa. O estudo segue a lista de verificação do *Transparent Reporting of Evaluations with Nonrandomized Designs* (TREND)^12^.

Este estudo é parte do macroprojeto “Diagnóstico Situacional das Infecções Sexualmente Transmissíveis no Contexto Amazônico”. A pesquisa foi embasada em duas etapas prévias do projeto: 1- Desenvolvimento e Validação de Cartilha em estudo anterior^13^: A cartilha educativa “Vamos falar sobre sífilis” foi desenvolvida com linguagem e expressões da comunidade ribeirinha para abordar transmissão, sintomas, diagnóstico, tratamento e prevenção^14^. 2- Construção e Validação do Questionário^15^ ^)^ “Avaliação do conhecimento de mulheres ribeirinhas sobre sífilis” que está em processo de publicação. Ambos os materiais foram submetidos à validação semântica com mulheres ribeirinhas da Ilha do Combu, Belém, Pará, e à validação de conteúdo por especialistas.

Dessa forma, o presente estudo realizou a intervenção educativa através da roda de conversa com leitura compartilhada da cartilha. 

### Local e período do estudo

Realizado na Estratégia Saúde da Família (ESF) da Ilha de Cotijuba, que está inserida no município de Belém, Pará. Possui distância de aproximadamente 22 km da capital belenense, tendo como principal acesso a via fluvial. A ilha tem uma população estimada de 9.000 habitantes e ocupa uma área de 16 km^2^. A população da ilha vive das atividades comerciais, da pesca artesanal, da agricultura familiar, do agroextrativismo e do turismo^16^. O estudo ocorreu entre os meses de abril a maio de 2024.

### Participantes

A população do estudo foi composta por mulheres ribeirinhas da Ilha de Cotijuba, Belém, Pará.

### Critérios de seleção

Mulheres ribeirinhas com idade maior ou igual a 18 anos, sem limite superior de idade, moradoras permanentes da Ilha de Cotijuba e alfabetizadas. O critério para inclusão de participantes alfabetizadas foi a autodeclaração, seguida da comprovação da capacidade de ler e assinar o Termo de Consentimento Livre e Esclarecido (TCLE) no momento do recrutamento. Os critérios de exclusão foram mulheres visitantes ou trabalhadoras não residentes da Ilha de Cotijuba. 

### Definição da amostra

As mulheres foram selecionadas de forma aleatória conforme a demanda, sendo, portanto, uma amostragem consecutiva, durante uma ação em saúde realizada pelos enfermeiros da ESF da Ilha de Cotijuba. O tamanho amostral foi estimado em 40 mulheres a partir da fórmula baseada no teste de Qui-quadrado de McNemar, utilizado em estudo prévio^11^. Nenhuma mulher negou participar do estudo. 

Para o cálculo, adotou-se os seguintes parâmetros: coeficiente de confiança de 95%, poder estatístico de 80%, proporção de pares que não sofreria alteração com a aplicação da intervenção educativa de 50% (adotou-se este valor por se considerar o desconhecimento deste parâmetro) e uma mudança de proporção de pelo menos 20% entre pares de observações para se rejeitar a hipótese de nulidade (ou seja, não haver diferença entre as proporções antes e após o desenvolvimento da intervenção educativa), onde Zα = 1,96; Zβ = 80%; PA = 0,2; qA =0,8; PD= 0,5. Após os cálculos, a amostra foi estimada em 40 participantes.

### Instrumento utilizado para a coleta das informações

Utilizou-se o questionário^15^ validado intitulado “Avaliação do conhecimento de mulheres ribeirinhas sobre sífilis”, desenvolvido pela pesquisadora como parte de sua dissertação. O questionário é composto por perguntas abertas e fechadas, estruturado em três partes: dados sociodemográficos (idade, escolaridade e estado civil), seis questões dicotômicas sobre fatores comportamentais e dez questões sobre o conhecimento acerca da sífilis. Esta última seção está subdividida em quatro tópicos principais: aspectos gerais da infecção, diagnóstico e prevenção, tratamento, e sífilis gestacional e congênita.

### Coleta de dados

O estudo foi conduzido em quatro etapas sequenciais: (i) Pré-teste, (ii) Intervenção educativa com a realização da roda de conversa com leitura compartilhada da cartilha “Vamos falar sobre Sífilis”, (iii) Pós-teste imediato, e (iv) Pós-teste 7 dias. As atividades foram conduzidas em locais protegidos e previamente acordados com as participantes, garantindo um ambiente seguro e confidencial para a discussão e a troca de experiências.

Inicialmente, 40 mulheres foram convidadas a participar do estudo na ESF da Ilha. Neste momento, a pesquisadora perguntava o nível de escolaridade das mulheres para avaliar se elas seriam capazes de ler o material educativo. Após a assinatura do TCLE, as participantes responderam a um questionário impresso (etapa i), com duração de 20 minutos, para avaliar o conhecimento prévio sobre a sífilis e caracterizar seu perfil sociodemográfico. As participantes foram orientadas a responder o questionário de acordo com seus conhecimentos prévios em relação a sífilis e que não poderiam fazer quaisquer tipos de consultas ou compartilhar as respostas com as demais. Cada questionário foi codificado para assegurar a rastreabilidade das respostas.

Na etapa ii, cada participante recebeu uma cópia impressa da cartilha “Vamos falar sobre Sífilis” e foi orientada a folheá-la e lê-la individualmente por 15 minutos. Em seguida, a pesquisadora enfermeira conduziu uma roda de conversa com a leitura compartilhada e manuseio da cartilha, bem como o esclarecimento de dúvidas. A duração total da roda de conversa foi de 30 minutos por grupo, sendo a mesma duração padronizada para os três grupos. Vale destacar que, durante a roda de conversa, algumas mulheres se sentiram à vontade e compartilharam experiências próprias e de amigos/familiares que foram diagnosticados e realizaram o tratamento para sífilis.

Na etapa iii, imediatamente após a intervenção, o mesmo questionário foi reaplicado para avaliar o conhecimento adquirido (pós-teste imediato), com 20 minutos para preenchimento. Ao final dessa etapa as cartilhas foram entregues às participantes para consulta em domicílio.

Por fim, a etapa iv consistiu na reaplicação do questionário via contato telefônico, sete dias após a intervenção^11^ ^,^ ^17^, com o objetivo de verificar a retenção do conhecimento. Apenas uma participante não foi localizada, resultando em uma amostra de 39 mulheres para esta fase do estudo.

### Tratamento e análise dos dados

Os dados coletados foram armazenados em uma planilha no *Microsoft Excel* ^*®*^ , versão 2019, e a análise estatística foi realizada no *software BioEstat* ^*®*^ , versão 5.0 e *Minitab* ^*®*^ . Inicialmente, realizou-se análise descritiva das características sociodemográficas e fatores comportamentais das mulheres ribeirinhas, descritas por meio de frequências e porcentagens. 

Os dados do conhecimento foram analisados inicialmente por estatística descritiva, com frequências absolutas e relativas e medidas de tendência central. Para avaliar se houve diferença na pontuação entre os grupos antes e após a intervenção, utilizou-se o teste de Shapiro-Wilk para identificar a normalidade das variáveis. Após o resultado demonstrar uma distribuição não normal, utilizou-se o teste não-paramétrico de Wilcoxon para comparar o conhecimento das mulheres ribeirinhas na fase pré-teste, pós-teste imediato e pós-teste 7 dias. Foi considerado p-valor significativo ≤ 0,05. O gráfico de *box-plot* foi utilizado para demonstrar a análise de significância estatística entre os grupos*.*

### Considerações éticas

Este estudo faz parte da última etapa de um macroprojeto aprovado pelo Comitê de Ética em Pesquisa do Instituto de Ciências da Saúde (ICS) da Universidade Federal do Pará (UFPA) sob CAAE nº 10821819.0.0000.0018 e parecer nº 6.301.468. O TCLE, por meio escrito, foi obtido de todas as participantes envolvidas no estudo. 

## Resultados

Quanto aos dados sociodemográficos, houve prevalência de mulheres ribeirinhas na faixa etária de 18 a 29 anos (n=14; 35,0%), casadas e/ou em união estável (n=23; 57,5%), com ensino fundamental (n=21; 52,5%). 

A maioria das mulheres já ouviu falar sobre sífilis (n=37; 92,5%) por meio de profissionais de saúde (n=25; 67,6%) ou assistiram a palestras (n=23; 57,5%). Já tinham feito exame (n=27; 67,5%) para diagnóstico de sífilis em algum momento, sendo o teste rápido (n=14; 51,9%) o método mais citado. Observou-se também o relato de tratamento anterior para sífilis (n=14; 35,0%) e a não utilização de preservativo durante as relações sexuais (n=23, 57,5%).

Quanto à avaliação do conhecimento sobre sífilis, a Tabela 1 apresenta o número de acertos referentes ao conhecimento sobre sífilis das mulheres ribeirinhas no pré-teste, pós-teste imediato e pós-teste 7 dias, considerando o total de 10 questões por teste. Destaca-se que na fase pós-teste 7 dias houve a participação de apenas 39 mulheres. Estes dados mostram que houve maior acerto sobre a temática nos dois momentos de pós-teste quando comparados com o pré-teste realizado no momento anterior à intervenção educativa com uso da cartilha. 

Ao comparar as medidas de tendência central dos acertos das mulheres, observou-se que a média no pré-teste foi de 6,25 (Mediana: 6,0; Desvio-Padrão: 1,46; Mínima: 3,0 e Máxima: 9,0), no pós-teste imediato foi de 8,95 (Mediana: 9,0; Desvio-Padrão: 0,87; Mínima: 7,0 e Máxima: 10,0) e no pós-teste 7 dias foi de 8,89 (Mediana: 9,0; Desvio-Padrão: 0,94; Mínima: 7,0 e Máxima: 10,0). Ressalta-se, assim, melhora na aquisição e na retenção do conhecimento sobre sífilis após a intervenção educativa. 

Quanto ao conhecimento sobre sífilis, de acordo com as dimensões de análise no pós-teste imediato, a mediana foi maior que no pré-teste, com significância estatística em todas as dimensões (p ≤ 0, 05), de acordo com a Tabela 2.

Ao realizar a comparação do conhecimento sobre sífilis, de acordo com as dimensões de análise, entre os dois momentos de pós-teste, observou-se que houve retenção de conhecimento mesmo após 7 dias da intervenção, conforme a Tabela 3. 

A Tabela 4 demonstra a comparação do conhecimento sobre sífilis no pré-teste e pós-teste 7 dias de acordo com as dimensões de análise. Observa-se significância estatística em todas as dimensões, o que sugere aumento do conhecimento de tais aspectos.

As mulheres ribeirinhas que participaram da intervenção educativa sobre sífilis com a cartilha obtiveram aumento do conhecimento sobre sífilis (p = 0,0001) no pós-teste imediato (Figura 1) e, ao se analisar o pós-teste imediato com o pós-teste 7 dias, observou-se que o conhecimento sobre sífilis adquirido se manteve por 7 dias. Na comparação do pré-teste e o pós-teste 7 dias, observou-se melhora e fixação do conhecimento (p = 0,0001).

**Tabela 1:** Número de acertos das mulheres ribeirinhas em relação ao conhecimento sobre sífilis, nos momentos pré-teste (n = 40), pós-teste imediato (n = 40), pós-teste 7 dias (n = 39). Belém, PA, Brasil, 2024

Mulher ribeirinha	Pré-Teste		Pós-Teste imediato			Pós-Teste 7 dias	
	n	%	n	%	n	%
1	5	50	10	100	10	100
2	6	60	10	100	10	100
3	5	50	9	90	8	80
4	3	30	9	90	10	100
5	7	70	7	70	9	90
6	8	80	8	80	8	80
7	8	80	10	100	10	100
8	7	70	8	80	8	80
9	5	50	9	90	8	80
10	6	60	9	90	9	90
11	3	30	8	80	8	80
12	8	80	9	90	10	100
13	8	80	9	90	8	80
14	8	80	9	90	8	80
15	9	90	10	100	10	100
16	6	60	7	70	7	70
17	6	60	10	100	10	100
18	5	50	8	80	8	80
19	7	70	8	80	9	90
20	5	50	8	80	8	80
21	7	70	10	100	9	90
22	6	60	9	90	9	90
23	7	70	9	90	9	90
24	7	70	9	100	10	100
25	6	60	9	90	9	90
26	5	50	9	100	10	10
27	7	70	9	100	9	90
28	8	80	8	80	8	80
29	5	50	10	100	10	100
30	8	80	9	100	8	80
31	5	50	9	90	9	90
32	5	50	9	90	8	80
33	6	70	10	100	10	100
34	6	60	10	100	7	70
35	7	70	9	90	9	90
36	4	40	9	90	8	80
37	5	50	10	100	9	90
38	8	80	9	90	10	100
39	8	80	10	100	10	100
40	8	80	10	100	-	-

**Tabela 2:** Comparação do conhecimento sobre sífilis das mulheres ribeirinhas (n = 40) no pré-teste e pós-teste imediato de acordo com cada questão do questionário. Belém, PA, Brasil, 2024

Questões do questionário	Pré-Teste		Pós-Teste imediato		p-valor*
	n	%	n	%	
**Aspectos gerais sobre a infecção pela sífilis**					
1. Etiologia	34	87,1	40	100	
2. Forma de transmissão da sífilis	35	89,7	40	100	
3. Sinais e sintomas	27	69,2	34	85,0	
Mediana da pontuação total dos aspectos gerais	2		3		<0,0001
**Diagnóstico e prevenção**					
4. Diagnóstico	23	57,5	34	85,0	
5. Prevenção	30	76,9	37	92,5	
Mediana da pontuação total do diagnóstico e prevenção	1		2		<0,0001
**Tratamento**					
6. Tratamento adequado	29	72,5	37	92,5	
7. Tratamento do parceiro	23	59,0	33	82,5	
Mediana da pontuação total das questões do tratamento	1		2		0,002
**Sífilis gestacional e congênita**					
8. Diagnóstico da sífilis gestacional	20	51,3	34	85,0	
9. Complicações da sífilis gestacional	25	62,5	37	92,5	
10. Complicações da sífilis congênita	27	69,2	36	90,0	
Mediana da pontuação total sobre sífilis gestacional e congênita	2		3		<0,0001

*p-valor - Teste de Wilcoxon

**Tabela 3:** Comparação do conhecimento sobre sífilis das mulheres ribeirinhas no pós-teste imediato (n = 40) e pós-teste 7 dias (n = 39) de acordo com cada questão do questionário. Belém, PA, Brasil, 2024

Questões do questionário	Pós-Teste imediato		Pós-Teste 7 dias		p-valor*
	n	%	n	%	
**Aspectos gerais sobre a infecção pela sífilis**				
1. Etiologia	40	100	39	100	
2. Forma de transmissão da sífilis	40	100	39	100	
3. Sinais e sintomas	34	85,0	32	82,1	
Mediana da pontuação total dos aspectos gerais	3		3		0,425
**Diagnóstico e prevenção**				
4. Diagnóstico	34	85,0	36	92,3	
5. Prevenção	37	92,5	36	92,3	
Mediana da pontuação total diagnóstico e prevenção	2		2		0,563
**Tratamento**				
6. Tratamento adequado	37	92,5	31	79,5	
7. Tratamento do parceiro	33	82,5	34	87,2	
Mediana da pontuação total do tratamento	2		2		0,463
**Sífilis gestacional e congênita**				
8. Diagnóstico da sífilis gestacional	34	85,0	35	89,7	
9. Complicações da sífilis gestacional	37	92,5	36	92,3	
10. Complicações da sífilis congênita	36	90,0	38	97,4	
Mediana da pontuação total sobre sífilis gestacional e congênita		3	3		0,207

*p-valor - Teste de Wilcoxon

**Tabela 4:** Comparação do conhecimento sobre sífilis das mulheres ribeirinhas no pré-teste (n = 40) e pós-teste 7 dias (n = 39) de acordo com cada questão do questionário. Belém, PA, Brasil, 2024

Questões do questionário	Pré Teste		Pós-Teste 7 dias		p-valor*
	n	%	n	%	
**Aspectos gerais sobre a infecção pela sífilis**				
1. Etiologia	34	87,1	39	100	
2. Forma de transmissão da sífilis	35	89,7	37	94,9	
3. Sinais e sintomas	27	69,2	32	82,1	
Mediana da pontuação total dos aspectos gerais	2		3		<0,0001
**Diagnóstico e prevenção**				
4. Diagnóstico	22	56,4	36	92,3	
5. Prevenção	30	76,9	36	92,3	
Mediana da pontuação total do diagnóstico e prevenção	1		2		<0,0001
**Tratamento**				
6. Tratamento adequado	28	71,8	31	7,5	
7. Tratamento do parceiro	22	56,4	34	87,2	
Mediana da pontuação total do tratamento	1		2		0,015
**Sífilis gestacional e congênita**				
8. Diagnóstico da sífilis gestacional	20	51,3	35	89,7	
9. Complicações da sífilis gestacional	24	61,5	36	92,3	
10. Complicações da sífilis congênita	27	69,2	38	97,4	
Mediana da pontuação total sobre sífilis gestacional e congênita	2		3		<0,0001

*p-valor - Teste de Wilcoxon

**Figura 1:** Análise de significância estatística entre os grupos no pré-teste, pós-teste imediato e pós-teste 7 dias

*p-valor - Teste de Wilcoxon

## Discussão

As mulheres participantes da intervenção educativa, jovens, com ensino fundamental e casadas ou em união estável, reflete o perfil de outras comunidades ribeirinhas do Brasil^6^ ^,^ ^18^. O conhecimento prévio limitado identificado no pré-teste entre as participantes (Mediana = 6 acertos) associado ao não uso de preservativos evidencia uma vulnerabilidade comportamental que eleva o risco de aquisição de IST. 

Sabe-se que o uso inadequado ou a ausência de preservativos é um fator de vulnerabilidade individual que deve ser abordado pelas equipes de saúde por meio de tecnologias educativas e uma linguagem acessível^10^ ^,^ ^19^. Além disso, embora algumas soubessem responder a determinadas perguntas sobre a doença, não souberam responder a questões mais específicas, o que reforça a necessidade de maior investimento em educação em saúde. 

Após a intervenção educativa, a mediana de acertos aumentou significativamente para 9 no pós-teste imediato e se manteve no pós-teste 7 dias. Essa aquisição de conhecimento é fundamental para a prevenção e a adesão ao tratamento da sífilis, uma vez que o desconhecimento pode intensificar a problemática da doença, dificultando a prevenção e a cura^11^. 

A eficácia da intervenção educativa com roda de conversa e leitura compartilhada da cartilha foi confirmada pela comparação entre o pré-teste e o pós-teste imediato, com aumento no número de acertos das questões, ressaltando a melhoria do conhecimento sobre sífilis. Um estudo quase-experimental^11^, que avaliou os efeitos do conhecimento, atitude e prática de gestantes antes e após uma intervenção educativa com leitura de cartilha sobre sífilis gestacional, identificou uma melhora estatisticamente significativa nesses três domínios após a leitura do material. Outros estudos quase-experimentais^20^ ^-^ ^22^ verificaram aumento no número de acertos das questões e do nível de conhecimento sobre determinada doença após intervenção educativa com atividades em grupo e vídeo educativo. 

Nossos achados estão alinhados com estudos internacionais que também verificaram a eficácia de intervenções educativas em contextos vulneráveis. Em Moçambique, um ensaio clínico randomizado controlado com mulheres em risco de HIV/AIDS utilizou uma intervenção didática psicossocial que resultou em um aumento significativo do conhecimento sobre os fatores de risco da doença e maior adesão a práticas preventivas^23^.

Em áreas rurais do Peru, intervenções com programas de educação nutricional familiar demonstraram um efeito positivo no nível de conhecimento e na adoção de hábitos alimentares mais saudáveis^24^. Tais resultados, em diferentes contextos geográficos, reforçam que as intervenções educativas são ferramentas eficazes para a promoção da saúde em comunidades vulneráveis. 

Embora o período de acompanhamento tenha sido curto (7 dias), a manutenção do conhecimento sobre sífilis após a intervenção sugere que a cartilha tem potencial para promover a retenção de informações, o que é um resultado promissor. Um estudo, que objetivou avaliar a efetividade de uma intervenção educativa sobre adesão ao tratamento e conhecimento sobre sífilis em puérperas com resultado positivo para a doença, identificou que o pós-teste revelou melhorias no conhecimento acerca da sífilis, com a pontuação pós-teste de 30 dias associada à melhor adesão ao tratamento^25^. Outro estudo sobre sífilis em gestantes verificou um aumento do conhecimento após a intervenção com cartilha, tanto no pós-teste imediato quanto no 7º dia^11^.

A transmissão vertical da sífilis continua a ser um desafio de saúde pública^26^_,_ com barreiras como a situação social desfavorável e a falta de conhecimento^27^_._ Diante disso, ações educativas voltadas para populações vulneráveis são estratégias eficazes para incentivar a adoção de práticas de promoção da saúde e colaborar para um maior controle da doença^28^.

Portanto, a implementação de estratégias como o uso de tecnologias acessíveis e intervenções educativas têm se mostrado eficaz para melhorar o conhecimento e os cuidados de saúde^11^ ^,^ ^19^ ^,^ ^25^. Acredita-se que intervenções educativas multimodais, com metodologia e materiais validados, podem melhorar o conhecimento e o autocuidado, especialmente entre os mais vulneráveis^29^.

Reitera-se que a intervenção educativa demonstrou ser eficaz ao melhorar o conhecimento sobre a sífilis entre as mulheres ribeirinhas, com resultados positivos mantidos por 7 dias. No entanto, a persistência de alguns erros no pós-teste sugere que o aprendizado é um processo contínuo e que são necessárias intervenções periódicas. Portanto, o papel da equipe de saúde é crucial para estabelecer um vínculo com a comunidade, facilitando a adoção de comportamentos saudáveis e garantindo que o conhecimento adquirido se traduza em ações concretas de prevenção e autocuidado, contribuindo para o controle da doença^11^ ^,^ ^25^ ^,^ ^27^. 

Na prática, a intervenção com a cartilha mostrou-se mais eficaz quando realizada em formato de roda de conversa e leitura compartilhada. Essa abordagem facilitou a interação, permitindo que as mulheres ribeirinhas se sentissem à vontade para relatar suas experiências e esclarecer dúvidas, o que reforça a importância de um método participativo. A intervenção teve baixo custo e a eficácia observada neste estudo justifica a realização de pesquisas futuras para explorar sua replicação e adaptabilidade em outros contextos, tanto em áreas rurais quanto urbanas. 

Como limitações do estudo, destaca-se o número amostral da intervenção educativa e sua realização em uma única comunidade, o que limita a capacidade de generalização. Dessa forma, torna-se relevante a realização de estudos futuros que possam ampliar o tamanho amostral de mulheres de diferentes comunidades ribeirinhas, assim como a realização de um estudo randomizado controlado para identificar o efeito da cartilha educativa na mudança de conhecimento sobre a sífilis. 

A principal contribuição do estudo é a evidência científica da eficácia da intervenção educativa, mediada pelo enfermeiro, com roda de conversa e leitura compartilhada da cartilha em uma população específica e pouco estudada devido especialmente à dificuldade de acesso. Ademais, isso não apenas aprimora as estratégias de educação em saúde para populações vulneráveis, mas também ressalta o potencial da enfermagem para liderar e implementar soluções de baixo custo e com impacto social, possibilitando a adesão a práticas preventivas e no enfrentamento de problemas de saúde pública como a sífilis.

## Conclusão

A intervenção educativa conduzida em formato de roda de conversa, com a leitura compartilhada da cartilha “Vamos falar sobre Sífilis”, mostrou-se eficaz na aquisição de conhecimento sobre sífilis entre mulheres ribeirinhas, evidenciada pela melhora significativa nos resultados do pós-teste. A manutenção do conhecimento por sete dias sugere o potencial da cartilha em atuar como um recurso de apoio duradouro. A linguagem clara e acessível do material, combinada à dinâmica da roda de conversa, demonstrou ser uma ferramenta valiosa para o processo de ensino-aprendizagem. A cartilha tem aplicabilidade comprovada e pode ser adaptada e utilizada por diversos profissionais da saúde e em diferentes contextos, tanto em comunidades rurais com acesso limitado quanto em áreas urbanas, auxiliando na prevenção da sífilis e na promoção do autocuidado.

## Declaração de Disponibilidade de Dados:

Os conjuntos de dados relacionados a este artigo estarão disponíveis mediante solicitação ao autor correspondente.

# Translation: Intervención educativa con cartilla sobre sífilis para mujeres en situación de vulnerabilidad sexual: estudio cuasiexperimental^3^

**DOI:** 10.1590/1518-8345.8010.4806

**Authors:** Vanessa Kelly Cardoso Estumano, Pedro Paulo Santos Nunes, Aline Maria Pereira Cruz Ramos, Marcia Helena Machado Nascimento, Guilherme Guarino de Moura Sá, Eliã Pinheiro Botelho, Cintia Yolette Urbano Pauxis Aben-Athar

**Affiliations:** 1 Universidade Federal do Pará, Belém, PA, Brasil.; 2 Universidade Federal do Pará, Faculdade de Enfermagem, Belém, PA, Brasil.; 3 Becario de la Coordenação de Aperfeiçoamento de Pessoal de Nível Superior (CAPES), Brasil.; 4 Universidade do Estado do Pará, Belém, PA, Brasil.; 5 Instituto Federal de Educação, Ciência e Tecnologia de Pernambuco, Pesqueira, PE, Brasil.

**Keywords:** Mujeres, Poblaciones Vulnerables, Vulnerabilidad Sexual, Sífilis, Estudio de Intervención, Enfermería.

## Abstract

**Objetivo:**

evaluar el conocimiento de mujeres ribereñas sobre sífilis antes y después de una intervención educativa con uso de cartilla.

**Método:**

estudio cuasiexperimental, comparativo, con diseño antes y después, realizado con 40 mujeres ribereñas mediante una rueda de conversación con lectura compartida de la cartilla. Para el análisis de los datos se utilizó la prueba no paramétrica de Wilcoxon.

**Resultados:**

la mayoría de las mujeres tenía entre 18 y 29 años, estaba casada o en unión estable y tenía educación primaria. La mayoría ya había oído hablar de la sífilis por medio de profesionales de la salud. Se realizaron pruebas para la detección de sífilis y ya habían recibido tratamiento previo para sífilis. Al comparar las puntuaciones del pretest y del postest inmediato, se observó un aumento del conocimiento sobre sífilis. Al analizar la fase postest inmediato frente al postest a 7 días, no hubo diferencias significativas, es decir, el conocimiento adquirido se mantuvo igual tras 7 días de la intervención educativa. Al comparar la fase pretest y la fase postest a 7 días, se observó una mejora del conocimiento.

**Conclusión:**

la intervención educativa mediante una rueda de conversación con lectura compartida de la cartilla se mostró eficaz para la adquisición de conocimiento sobre sífilis entre mujeres ribereñas, evidenciada por la mejora significativa del conocimiento en los resultados del postest.

## Destacados

**(1)** Las mujeres presentaron aumento y mantenimiento del conocimiento sobre sífilis. **(2)** La intervención con la cartilla y la rueda de conversación fue eficaz para incrementar el conocimiento. **(3)** La cartilla es una tecnología facilitadora del proceso de enseñanza-aprendizaje. **(4)** La rueda de conversación con lectura compartida de la cartilla aumentó el conocimiento.

## Introducción

La sífilis es una Infección de Transmisión Sexual (IST) considerada un problema de salud pública por ser una afección de magnitud global^1^. A pesar de los avances en el acceso al diagnóstico mediante pruebas rápidas, que facilitan la identificación y el tratamiento, la reducción de casos de la enfermedad aún no se ha observado, lo que apunta a lagunas en las estrategias de control y prevención^2^.

Ante este escenario, la persistencia de los casos de sífilis resalta la necesidad urgente de acciones de combate para que las metas establecidas en la Agenda 2030 de la Organización de las Naciones Unidas (ONU), en cuanto a la reducción de IST, entre ellas la sífilis, sean alcanzadas^3^ ^-^ ^4^. En ese contexto, las iniciativas de enfrentamiento deben ir más allá del diagnóstico y el tratamiento, y necesitan estar fundamentadas en el movimiento social y en la planificación en salud^5^. Es fundamental que estas acciones se dirijan específicamente a las comunidades y poblaciones vulnerables que enfrentan un acceso dificultado a la salud.

Esa dificultad de acceso, característica de poblaciones como las ribereñas, se ve amplificada en los vastos y complejos territorios brasileños, como en la Amazonía. En esas regiones, las grandes distancias geográficas y la desigualdad social perpetúan la transmisión de la sífilis entre en la población adulta^2^ ^,^ ^6^, y la falta de conocimiento sobre las ISTs se identifica como uno de los factores que contribuyen a la infección por *Treponema pallidum*, el agente etiológico de la enfermedad^2^.

En este contexto de vulnerabilidad ampliada, las poblaciones ribereñas de la Amazonía, foco de este estudio, tienen su rutina moldeada por la influencia de los ríos. La economía, basada en la pesca, el extractivismo vegetal y, en ocasiones, en actividades turísticas, convive con una infraestructura precaria. Esta realidad está marcada por la falta de saneamiento básico, el acceso limitado a la energía eléctrica y el acceso restringido a servicios de salud y políticas públicas^6^ ^-^ ^7^.

Aunque marcadas por la centralidad de la presencia femenina y por el papel esencial de la mujer en la configuración de la comunidad, las mujeres ribereñas de la Amazonía enfrentan vulnerabilidades sociales significativas^8^. Tal escenario, sumado al bajo conocimiento y a comportamientos sexuales de riesgo, las vuelve particularmente susceptibles a desenlaces desfavorables para la salud, especialmente en lo que atañe a la salud sexual^6^ ^,^ ^9^ ^-^ ^10^.

Así, el estilo de vida y el escenario de estas poblaciones exigen una respuesta diferenciada en el enfrentamiento de la sífilis, distinta del abordaje dirigido a la población en general. Por lo tanto, debe garantizarse a estas poblaciones vulnerables el acceso a información de calidad, mediante acciones de promoción de la salud y prevención de la sífilis realizada por el equipo de salud^6^.

Para que estas acciones sean verdaderamente eficaces, es imperativo que atiendan a las peculiaridades regionales, así como a los comportamientos y actitudes^2^ de los habitantes de diferentes contextos vulnerables de Brasil, ya sean de los campos, ríos o selvas. El equipo de salud debe, por lo tanto, realizar actividades de educación en salud, en grupo o individualmente, utilizando un lenguaje accesible y dirigido a las características comportamentales locales^10^.

En ese sentido, el uso de tecnologías validadas y de fácil acceso, como las cartillas, se muestra fundamental, pues además de auxiliar en el empoderamiento del individuo, permite que este actúe como multiplicador, presentando el material a otros individuos de la comunidad. De esta forma, las cartillas son materiales útiles para la descripción de temas relacionados con la salud, siendo posible su utilización como instrumento de promoción de la salud, facilitadora del proceso educativo y mejora del conocimiento^11^.

Teniendo en cuenta la necesidad de estrategias de educación en salud para poblaciones vulnerables, y reconociendo la eficacia de tecnologías educativas validadas, este estudio tuvo como objetivo evaluar el conocimiento de mujeres ribereñas sobre sífilis antes y después de una intervención educativa con uso de cartilla.

## Método

### Diseño del estudio

Este es un estudio cuasiexperimental, comparativo, con un diseño antes y después (pre y postest). Las participantes sirvieron como sus propios controles, siendo evaluados antes y después de la actividad educativa. El estudio sigue la lista de verificación del *Transparent Reporting of Evaluations with Nonrandomized Designs* (TREND)^12^.

Este estudio forma parte del macroproyecto “Diagnóstico Situacional de las Infecciones de Transmisión Sexual en el Contexto Amazónico”. La investigación se basó en dos etapas previas del proyecto: 1- Desarrollo y validación de cartilla en estudio anterior^13^: la cartilla educativa “Vamos a hablar sobre sífilis” fue desarrollada con lenguaje y expresiones de la comunidad ribereña para abordar transmisión, síntomas, diagnóstico, tratamiento y prevención^14^. 2- Construcción y validación del cuestionario^15^ “Evaluación del conocimiento de mujeres ribereñas sobre sífilis”, que se encuentra en proceso de publicación. Ambos materiales fueron sometidos a validación semántica con mujeres ribereñas de la Ilha do Combu, Belém, Pará, y a validación de contenido por especialistas.

De esta forma, el presente estudio realizó la intervención educativa mediante una rueda de conversación con lectura compartida de la cartilla. 

### Lugar y período del estudio

Realizado en la *Estratégia Saúde da Família* (ESF) de la Isla de Cotijuba, ubicada en el municipio de Belém, Pará. Se encuentra a una distancia de aproximadamente 22 km de la capital belenense, teniendo como principal acceso la vía fluvial. La isla tiene una población estimada de 9.000 habitantes y ocupa un área de 16 km². La población de la isla vive de actividades comerciales, de la pesca artesanal, de la agricultura familiar, del agroextractivismo y del turismo^16^. El estudio se llevó a cabo entre los meses de abril y mayo de 2024.

### Participantes

La población del estudio estuvo compuesta por mujeres ribereñas de la Isla de Cotijuba, Belém, Pará.

### Criterios de selección

Mujeres ribereñas con edad igual o superior a 18 años, sin límite superior de edad, residentes permanentes de la Isla de Cotijuba y alfabetizadas. El criterio para la inclusión de participantes alfabetizados fue la autodeclaración, seguida de la comprobación de la capacidad de leer y firmar el Consentimiento Informado (CI) en el momento del reclutamiento. Los criterios de exclusión fueron mujeres visitantes o trabajadoras no residentes de la Isla de Cotijuba. 

### Definición de la muestra

Las mujeres fueron seleccionadas de forma aleatoria conforme a la demanda, constituyendo, por lo tanto, un muestreo consecutivo, durante una acción en salud realizada por los enfermeros de la ESF de la Isla de Cotijuba. El tamaño muestral se estimó en 40 mujeres a partir de la fórmula basada en la prueba de chi-cuadrado de McNemar, utilizada en un estudio previo^11^. Ninguna mujer rechazó participar en el estudio.

Para el cálculo, se adoptaron los siguientes parámetros: coeficiente de confianza del 95%, poder estadístico del 80%, proporción de pares que no sufriría alteración con la aplicación de la intervención educativa del 50% (se adoptó este valor por considerarse desconocido este parámetro) y un cambio de proporción de por lo menos 20% entre pares de observaciones para rechazar la hipótesis de nulidad (es decir, no haber diferencia entre las proporciones antes y después del desarrollo de la intervención educativa), donde Zα = 1,96; Zβ = 80%; PA = 0,2; qA = 0,8; PD = 0,5. Tras los cálculos, la muestra se estimó en 40 participantes.

### Instrumento utilizado para la recolección de la información

Se utilizó el cuestionario^15^ validado titulado “Evaluación del conocimiento de mujeres ribereñas sobre sífilis”, desarrollado por la investigadora como parte de su disertación. El cuestionario está compuesto por preguntas abiertas y cerradas, estructurado en tres partes: datos sociodemográficos (edad, escolaridad y estado civil), seis preguntas dicotómicas sobre factores conductuales y diez preguntas sobre el conocimiento acerca de la sífilis. Esta última sección está subdividida en cuatro temas principales: aspectos generales de la infección, diagnóstico y prevención, tratamiento, y sífilis gestacional y congénita.

### Recolección de datos

El estudio se condujo en cuatro etapas secuenciales: (i) pretest, (ii) intervención educativa con la realización de la rueda de conversación con lectura compartida de la cartilla “Vamos a hablar sobre Sífilis”, (iii) postest inmediato y (iv) postest a 7 días. Las actividades fueron conducidas en lugares protegidos y previamente acordados con las participantes, garantizando un ambiente seguro y confidencial para la discusión y el intercambio de experiencias.

Inicialmente, 40 mujeres fueron invitadas a participar del estudio en la ESF de la isla. En ese momento, la investigadora preguntaba el nivel de escolaridad de las mujeres para evaluar si serían capaces de leer el material educativo. Tras la firma del CI, las participantes respondieron un cuestionario impreso (etapa i), con una duración de 20 minutos, para evaluar el conocimiento previo sobre la sífilis y caracterizar su perfil sociodemográfico. Las participantes fueron orientadas a responder el cuestionario de acuerdo con sus conocimientos previos en relación con la sífilis y a no realizar ningún tipo de consulta ni compartir las respuestas con las demás. Cada cuestionario fue codificado para asegurar la rastreabilidad de las respuestas.

En la etapa ii, cada participante recibió una copia impresa de la cartilla “Vamos a hablar sobre Sífilis” y fue orientada a hojearla y leerla individualmente durante 15 minutos. A continuación, la investigadora enfermera condujo una rueda de conversación con la lectura compartida y el manejo de la cartilla, así como el esclarecimiento de dudas. La duración total de la rueda de conversación fue de 30 minutos por grupo, siendo la misma duración estandarizada para los tres grupos. Cabe destacar que, durante la rueda de conversación, algunas mujeres se sintieron cómodas y compartieron experiencias propias y de amigos/familiares que fueron diagnosticados y realizaron el tratamiento para sífilis.

En la etapa iii, inmediatamente después de la intervención, el mismo cuestionario se volvió a aplicar para evaluar el conocimiento adquirido (postest inmediato), con 20 minutos para el llenado. Al final de esta etapa, las cartillas fueron entregadas a las participantes para consulta en el domicilio.

Por último, la etapa iv consistió en la nueva aplicación del cuestionario mediante contacto telefónico, siete días después de la intervención^11^ ^,^ ^17^, con el objetivo de verificar la retención del conocimiento. Solo una participante no fue localizada, resultando en una muestra de 39 mujeres para esta fase del estudio.

### Tratamiento y análisis de los datos

Los datos recolectados fueron almacenados en una hoja de cálculo en *Microsoft Excel* ^*®*^ , versión 2019, y el análisis estadístico se realizó en el *software BioEstat* ^*®*^ , versión 5.0, y *Minitab* ^*®*^ . Inicialmente, se efectuó un análisis descriptivo de las características sociodemográficas y de los factores conductuales de las mujeres ribereñas, descritas por medio de frecuencias y porcentajes.

Los datos sobre el conocimiento fueron analizados inicialmente mediante estadística descriptiva, con frecuencias absolutas y relativas y medidas de tendencia central. Para evaluar si hubo diferencia en la puntuación entre los grupos antes y después de la intervención, se utilizó la prueba de Shapiro-Wilk para identificar la normalidad de las variables. Al evidenciarse una distribución no normal, se empleó la prueba no paramétrica de Wilcoxon para comparar el conocimiento de las mujeres ribereñas en la fase pretest, postest inmediato y postest a 7 días. Se consideró valor p significativo ≤ 0,05. El *box-plot* se utilizó para demostrar el análisis de significancia estadística entre los grupos.

### Consideraciones éticas

Este estudio forma parte de la última etapa de un macroproyecto aprobado por el Comité de Ética en Investigación del Instituto de Ciencias de la Salud (ICS) de la *Universidade Federal do Pará* (UFPA) bajo CAAE n.º 10821819.0.0000.0018 y dictamen n.º 6.301.468. El consentimiento informado por medio escrito, fue obtenido de todas las participantes involucradas en el estudio. 

## Resultados

En cuanto a los datos sociodemográficos, hubo prevalencia de mujeres ribereñas en el grupo etario de 18 a 29 años (n=14; 35,0%), casadas y/o en unión estable (n=23; 57,5%), con educación primaria (n=21; 52,5%).

La mayoría de las mujeres ya había oído hablar sobre sífilis (n=37; 92,5%) por medio de profesionales de la salud (n=25; 67,6%) o asistieron a charlas (n=23; 57,5%). Ya se habían realizado prueba (n=27; 67,5%) para diagnóstico de sífilis en algún momento, siendo la prueba rápida (n=14; 51,9%) el método más citado. Se observó también el relato de tratamiento previo para sífilis (n=14; 35,0%) y la no utilización de preservativo durante las relaciones sexuales (n=23; 57,5%).

En cuanto a la evaluación del conocimiento sobre sífilis, la Tabla 1 presenta el número de aciertos referentes al conocimiento sobre sífilis de las mujeres ribereñas en el pretest, postest inmediato y postest a 7 días, considerando el total de 10 preguntas por prueba. Se destaca que en la fase postest a 7 días hubo la participación de apenas 39 mujeres. Estos datos muestran que hubo mayor acierto sobre la temática en los dos momentos de postest cuando se comparan con el pretest realizado en el momento anterior a la intervención educativa con uso de la cartilla. 

Al comparar las medidas de tendencia central de los aciertos de las mujeres, se observó que la media en el pretest fue de 6,25 (Mediana: 6,0; Desviación estándar: 1,46; Mínima: 3,0 y Máxima: 9,0), en el postest inmediato fue de 8,95 (Mediana: 9,0; Desviación estándar: 0,87; Mínima: 7,0 y Máxima: 10,0) y en el postest a 7 días fue de 8,89 (Mediana: 9,0; Desviación estándar: 0,94; Mínima: 7,0 y Máxima: 10,0). Se resalta, así, una mejora en la adquisición y en la retención del conocimiento sobre sífilis tras la intervención educativa.

El conocimiento sobre sífilis, de acuerdo con las dimensiones de análisis, en el postest inmediato presentó una mediana mayor que en el pretest, con significancia estadística en todas las dimensiones (p ≤ 0,05), de acuerdo con la Tabla 2.

Al realizar la comparación del conocimiento sobre sífilis, de acuerdo con las dimensiones de análisis, entre los dos momentos de postest, se observó que hubo retención del conocimiento incluso después de 7 días de la intervención, conforme a la Tabla 3.

La Tabla 4 demuestra la comparación del conocimiento sobre sífilis en el pretest y postest a 7 días de acuerdo con las dimensiones de análisis. Se observa significancia estadística en todas las dimensiones, lo que sugiere un aumento en el conocimiento de tales aspectos.

Las mujeres ribereñas que participaron en la intervención educativa sobre sífilis con la cartilla obtuvieron un aumento del conocimiento sobre sífilis (p = 0,0001) en el postest inmediato (Figura 1) y, al analizar el postest inmediato con el postest a 7 días, se observó que el conocimiento adquirido se mantuvo durante 7 días. En la comparación entre el pretest y el postest a 7 días, se observó mejora y fijación del conocimiento (p = 0,0001).

**Tabla 1:** Número de aciertos de las mujeres ribereñas en relación con el conocimiento sobre sífilis, en los momentos pretest (n = 40), postest inmediato (n = 40), postest a 7 días (n = 39). Belém, PA, Brasil, 2024

Mujer ribereña	Pretest		Postest inmediato		Postest 7 días	
	n	%	n	%	n	%
1	5	50	10	100	10	100
2	6	60	10	100	10	100
3	5	50	9	90	8	80
4	3	30	9	90	10	100
5	7	70	7	70	9	90
6	8	80	8	80	8	80
7	8	80	10	100	10	100
8	7	70	8	80	8	80
9	5	50	9	90	8	80
10	6	60	9	90	9	90
11	3	30	8	80	8	80
12	8	80	9	90	10	100
13	8	80	9	90	8	80
14	8	80	9	90	8	80
15	9	90	10	100	10	100
16	6	60	7	70	7	70
17	6	60	10	100	10	100
18	5	50	8	80	8	80
19	7	70	8	80	9	90
20	5	50	8	80	8	80
21	7	70	10	100	9	90
22	6	60	9	90	9	90
23	7	70	9	90	9	90
24	7	70	9	100	10	100
25	6	60	9	90	9	90
26	5	50	9	100	10	10
27	7	70	9	100	9	90
28	8	80	8	80	8	80
29	5	50	10	100	10	100
30	8	80	9	100	8	80
31	5	50	9	90	9	90
32	5	50	9	90	8	80
33	6	70	10	100	10	100
34	6	60	10	100	7	70
35	7	70	9	90	9	90
36	4	40	9	90	8	80
37	5	50	10	100	9	90
38	8	80	9	90	10	100
39	8	80	10	100	10	100
40	8	80	10	100	-	-

**Tabla 2:** Comparación del conocimiento sobre sífilis de las mujeres ribereñas (n = 40) en el pretest y postest inmediato de acuerdo con cada pregunta del cuestionario. Belém, PA, Brasil, 2024

Preguntas del cuestionario	Pretest		Postest inmediato		p-valor*
	n	%	n	%	
**Aspectos generales sobre la infección por sífilis**					
1. Etiología	34	87,1	40	100	
2. Forma de transmisión de la sífilis	35	89,7	40	100	
3. Signos y síntomas	27	69,2	34	85,0	
Mediana de la puntuación total de los aspectos generales	2		3		<0,0001
**Diagnóstico y prevención**					
4. Diagnóstico	23	57,5	34	85,0	
5. Prevención	30	76,9	37	92,5	
Mediana de la puntuación total del diagnóstico y la prevención	1		2		<0,0001
**Tratamiento**					
6. Tratamiento adecuado	29	72,5	37	92,5	
7. Tratamiento de la pareja	23	59,0	33	82,5	
Mediana de la puntuación total de las preguntas de tratamiento	1		2		0,002
**Sífilis gestacional y congénita**					
8. Diagnóstico de la sífilis gestacional	20	51,3	34	85,0	
9. Complicaciones de la sífilis gestacional	25	62,5	37	92,5	
10. Complicaciones de la sífilis congénita	27	69,2	36	90,0	
Mediana de la puntuación total sobre sífilis gestacional y congénita	2		3		<0,0001

*p-valor - Prueba de Wilcoxon

**Tabla 3:** Comparación del conocimiento sobre sífilis de las mujeres ribereñas en el postest inmediato (n = 40) y postest a 7 días (n = 39) de acuerdo con cada pregunta del cuestionario. Belém, PA, Brasil, 2024

Preguntas del cuestionario	Postest inmediato		Postest a 7 días		p-valor*
	n	%	n	%	
**Aspectos generales sobre la infección por sífilis**				
1. Etiología	40	100	39	100	
2. Forma de transmisión de la sífilis	40	100	39	100	
3. Signos y síntomas	34	85,0	32	82,1	
Mediana de la puntuación total de los aspectos generales	3		3		0,425
**Diagnóstico y prevención**				
4. Diagnóstico	34	85,0	36	92,3	
5. Prevención	37	92,5	36	92,3	
Mediana de la puntuación total del diagnóstico y la prevención	2		2		0,563
**Tratamiento**				
6. Tratamiento adecuado	37	92,5	31	79,5	
7. Tratamiento de la pareja	33	82,5	34	87,2	
Mediana de la puntuación total del tratamiento	2		2		0,463
**Sífilis gestacional y congénita**				
8. Diagnóstico de la sífilis gestacional	34	85,0	35	89,7	
9. Complicaciones de la sífilis gestacional	37	92,5	36	92,3	
10. Complicaciones de la sífilis congénita	36	90,0	38	97,4	
Mediana de la puntuación total sobre sífilis gestacional y congénita		3	3		0,207

*p-valor - Prueba de Wilcoxon

**Tabla 4:** Comparación del conocimiento sobre sífilis de las mujeres ribereñas en el pretest (n = 40) y postest a 7 días (n = 39) de acuerdo con cada pregunta del cuestionario. Belém, PA, Brasil, 2024

Preguntas del cuestionario	Pretest		Postest a 7 días		p-valor*
	n	%	n	%	
**Aspectos generales sobre la infección por sífilis**				
1. Etiología	34	87,1	39	100	
2. Forma de transmisión de la sífilis	35	89,7	37	94,9	
3. Signos y síntomas	27	69,2	32	82,1	
Mediana de la puntuación total de los aspectos generales	2		3		<0,0001
**Diagnóstico y prevención**				
4. Diagnóstico	22	56,4	36	92,3	
5. Prevención	30	76,9	36	92,3	
Mediana de la puntuación total del diagnóstico y la prevención	1		2		<0,0001
**Tratamiento**				
6. Tratamiento adecuado	28	71,8	31	7,5	
7. Tratamiento de la pareja	22	56,4	34	87,2	
Mediana de la puntuación total del tratamiento	1		2		0,015
**Sífilis gestacional y congénita**					
8. Diagnóstico de la sífilis gestacional	20	51,3	35	89,7	
9. Complicaciones de la sífilis gestacional	24	61,5	36	92,3	
10. Complicaciones de la sífilis congénita	27	69,2	38	97,4	
Mediana de la puntuación total sobre sífilis gestacional y congénita	2		3		<0,0001

*p-valor - Prueba de Wilcoxon

**Figura 1:** Análisis de significancia estadística entre los grupos en el pretest, postest inmediato y postest a 7 días

*p-valor - Prueba de Wilcoxon

## Discusión

Las mujeres participantes de la intervención educativa, jóvenes, con educación primaria y casadas o en unión estable, reflejan el perfil de otras comunidades ribereñas de Brasil^6^ ^,^ ^18^. El conocimiento previo limitado identificado en el pretest entre las participantes (Mediana = 6 aciertos), asociado al no uso de preservativos, evidencia una vulnerabilidad conductual que eleva el riesgo de adquisición de IST.

Se sabe que el uso inadecuado o la ausencia de preservativos es un factor de vulnerabilidad individual que debe ser abordado por los equipos de salud mediante tecnologías educativas y un lenguaje accesible^10^ ^,^ ^19^. Además, aunque algunas sabían responder ciertas preguntas sobre la enfermedad, no supieron responder cuestiones más específicas, lo que refuerza la necesidad de mayor inversión en educación en salud.

Tras la intervención educativa, la mediana de aciertos aumentó significativamente a 9 en el postest inmediato y se mantuvo en el postest a 7 días. Esta adquisición de conocimiento es fundamental para la prevención y la adherencia al tratamiento de la sífilis, ya que el desconocimiento puede intensificar la problemática de la enfermedad, dificultando la prevención y la curación^11^.

La eficacia de la intervención educativa con rueda de conversación y lectura compartida de la cartilla fue confirmada por la comparación entre el pretest y el postest inmediato, con aumento en el número de aciertos de las preguntas, resaltando la mejora del conocimiento sobre sífilis. Un estudio cuasiexperimental^11^, que evaluó los efectos en conocimiento, actitud y práctica de gestantes antes y después de una intervención educativa con lectura de cartilla sobre sífilis gestacional, identificó una mejora estadísticamente significativa en esos tres dominios tras la lectura del material. Otros estudios cuasiexperimentales^20^ ^-^ ^22^ verificaron aumento en el número de aciertos de las preguntas y del nivel de conocimiento sobre determinada enfermedad después de una intervención educativa con actividades grupales y video educativo.

Nuestros hallazgos están alineados con estudios internacionales que también verificaron la eficacia de intervenciones educativas en contextos vulnerables. En Mozambique, un ensayo clínico aleatorizado y controlado con mujeres en riesgo de VIH/SIDA utilizó una intervención didáctica psicosocial que resultó en un aumento significativo del conocimiento sobre los factores de riesgo de la enfermedad y mayor adherencia a prácticas preventivas^23^.

En áreas rurales de Perú, intervenciones con programas de educación nutricional familiar demostraron un efecto positivo en el nivel de conocimiento y en la adopción de hábitos alimentarios más saludables^24^. Tales resultados, en diferentes contextos geográficos, refuerzan que las intervenciones educativas son herramientas poderosas para la promoción de la salud en comunidades vulnerables.

Aunque el período de seguimiento haya sido corto (7 días), el mantenimiento del conocimiento sobre sífilis después de la intervención sugiere que la cartilla tiene potencial para promover la retención de información, lo que es un resultado prometedor. Un estudio que tuvo como objetivo evaluar la efectividad de una intervención educativa sobre adherencia al tratamiento y conocimiento sobre sífilis en puérperas con resultado positivo para la enfermedad identificó que el postest reveló mejoras en el conocimiento acerca de la sífilis, con la puntuación del postest a 30 días asociada a una mejor adherencia al tratamiento^25^. Otro estudio sobre sífilis en gestantes verificó un aumento del conocimiento tras la intervención con cartilla, tanto en el postest inmediato como en el 7º día^11^.

La transmisión vertical de la sífilis continúa siendo un desafío de salud pública^26^, con barreras como la situación social desfavorable y la falta de conocimiento^27^. Ante ello, las acciones educativas dirigidas a poblaciones vulnerables son estrategias eficaces para incentivar la adopción de prácticas de promoción de la salud y colaborar en un mayor control de la enfermedad^28^.

Por lo tanto, la implementación de estrategias como el uso de tecnologías accesibles e intervenciones educativas se ha mostrado eficaz para mejorar el conocimiento y los cuidados de salud^11^ ^,^ ^19^ ^,^ ^25^. Se considera que las intervenciones educativas multimodales, con metodología y materiales validados, pueden mejorar el conocimiento y el autocuidado, especialmente entre los más vulnerables^29^.

Se reitera que la intervención educativa demostró ser eficaz al mejorar el conocimiento sobre la sífilis entre las mujeres ribereñas, con resultados positivos mantenidos por 7 días. No obstante, la persistencia de algunos errores en el postest sugiere que el aprendizaje es un proceso continuo y que son necesarias intervenciones periódicas. Por lo tanto, el papel del equipo de salud es crucial para establecer un vínculo con la comunidad, facilitando la adopción de comportamientos saludables y garantizando que el conocimiento adquirido se traduzca en acciones concretas de prevención y autocuidado, contribuyendo al control de la enfermedad^11^ ^,^ ^25^ ^,^ ^27^.

En la práctica, la intervención con la cartilla se mostró más eficaz cuando se realizó en formato de rueda de conversación y lectura compartida. Este enfoque facilitó la interacción, permitiendo que las mujeres ribereñas se sintieran cómodas para relatar sus experiencias y aclarar dudas, lo que refuerza la importancia de un método participativo. La intervención tuvo bajo costo y la eficacia observada en este estudio justifica la realización de investigaciones futuras para explorar su replicación y adaptabilidad en otros contextos, tanto en áreas rurales como urbanas.

Como limitaciones del estudio, se destaca el tamaño muestral de la intervención educativa y su realización en una única comunidad, lo que limita la capacidad de generalización. De este modo, resulta relevante la realización de estudios futuros que puedan ampliar el tamaño muestral de mujeres de diferentes comunidades ribereñas, así como la realización de un estudio aleatorizado y controlado para identificar el efecto de la cartilla educativa en el cambio de conocimiento sobre la sífilis.

La principal contribución del estudio es la evidencia científica de la eficacia de la intervención educativa, mediada por el enfermero, con rueda de conversación y lectura compartida de la cartilla en una población específica y poco estudiada debido, especialmente, a la dificultad de acceso. Además, esto no solo mejora las estrategias de educación en salud para poblaciones vulnerables, sino que también resalta el potencial de la enfermería para liderar e implementar soluciones de bajo costo y con impacto social, posibilitando la adhesión a prácticas preventivas y el enfrentamiento de problemas de salud pública, como la sífilis.

## Conclusión

La intervención educativa conducida, en formato de rueda de conversación, con la lectura compartida de la cartilla “Vamos a hablar sobre Sífilis”, se mostró eficaz en la adquisición de conocimiento sobre sífilis entre mujeres ribereñas, evidenciada por la mejora significativa en los resultados del postest. El mantenimiento del conocimiento por siete días sugiere el potencial de la cartilla para actuar como un recurso de apoyo duradero. El lenguaje claro y accesible del material, combinado con la dinámica de la rueda de conversación, demostró ser una herramienta valiosa para el proceso de enseñanza-aprendizaje. La cartilla tiene aplicabilidad comprobada y puede ser adaptada y utilizada por diversos profesionales de la salud y en diferentes contextos, tanto en comunidades rurales con acceso limitado como en áreas urbanas, contribuyendo a la prevención de la sífilis y a la promoción del autocuidado.

## Declaración de Disponibilidad de Datos:

Los conjuntos de datos relacionados con este artículo estarán disponibles previa solicitud al autor correspondiente.
